# Spider Web-Inspired Lightweight Membrane-Type Acoustic Metamaterials for Broadband Low-Frequency Sound Isolation

**DOI:** 10.3390/polym13071146

**Published:** 2021-04-02

**Authors:** Heyuan Huang, Ertai Cao, Meiying Zhao, Sagr Alamri, Bing Li

**Affiliations:** 1 School of Aeronautics, Northwestern Polytechnical University, Xi’an 710072, China; huangheyuan@nwpu.edu.cn (H.H.); caoertai@live.cn (E.C.); zhaomeiying@nwpu.edu.cn (M.Z.); 2 Aircraft Strength Research Institute, Aviation Industries of China, Xi’an 710072, China; 3 Department of Mechanical Engineering, College of Engineering, King Khalid University, P.O. Box 394, Abha 61421, Saudi Arabia; salamri@kku.edu.sa

**Keywords:** membrane-type acoustic metamaterials, bio-inspired structures, polymeric membrane, anti-resonance, low-frequency sound isolation, sound transmission loss

## Abstract

Membrane-type acoustic metamaterial (MAM) has exhibited superior sound isolation properties, as well as thin and light characteristics. However, the anti-resonance modes of traditional MAMs are generated intermittently in a wide frequency range causing discontinuities in the anti-resonance modes. Achieving broadband low-frequency sound attenuation with lightweight MAM design is still a pivotal research aspect. Here, we present a strategy to realize wide sound-attenuation bands in low frequency range by introducing the design concept of bionic configuration philosophy into the MAM structures. Built by a polymeric membrane and a set of resonators, two kinds of MAM models are proposed based on the insight of a spider web topology. The sound attenuation performance and physical mechanisms are numerically and experimentally investigated. Multi-state anti-resonance modes, induced by the coupling of the bio-inspired arrangement and the host polymer film, are systematically explored. Significant sound attenuation is numerically and experimentally observed in both the lightweight bio-inspired designs. Remarkably, compared with a traditional MAM configuration, a prominent enhancement in both attenuation bandwidth and weight-reduction performance is verified. In particular, the bio-inspired MAM Model I exhibits a similar isolation performance as the reference model, but the weight is reduced by nearly half. The bio-inspired Model II broadens the sound attenuation bandwidth greatly; meanwhile, it retains a lighter weight design. The proposed bio-inspired strategies provide potential ways for designing sound isolation devices with both high functional and lightweight performance.

## 1. Introduction

Ever-increasing requirements and higher demands for noise and vibration suppression have attracted abundant effort to design novel structures and materials that are lightweight, yet with exceptional sound isolation/attenuation performance. However, achieving low-frequency noise isolation in lightweight structures is still an existing challenge, which lies in the fact of the mass law that poor sound absorption performance corresponding to lightweight design [1]. Traditional noise suppression approaches mostly adopt increasing structural size or weight to improve the blocking and attenuation effects on airborne sound. However, traditional noise barriers, such as rubber, felt, sponge, etc., do not meet the volume or performance requirements of modern advanced engineering for highly efficient noise isolation, especially in low-frequency range, due to the structural limitation to the long wavelength [2,3,4].

Recently, the concept of acoustic metamaterials has opened a new route to low-frequency noise isolation with compact and lightweight structures, which can realize unique bandgap effects for effectively blocking wave propagation at the corresponding frequencies based on locally resonant behaviors [5,6,7]. Such artificial structures have realized a flurry of abnormal dynamic properties, including single/double negative/zero mass-density or modulus etc. [8,9,10,11,12,13,14,15], relying on engineered subwavelength microstructures rather than the chemical compositions. A rich variety of extraordinary acoustic wave manipulations, such as negative refraction [13,16], acoustic cloaking [16], unidirectional transmission [17] and so forth, have been enabled by such an innovative design philosophy. Various types of local-resonance-based acoustic metamaterials with binary/multi-phase materials [2,3,4,15,18,19,20] have been proposed and developed for noise attenuation since the first design built by Liu et al. by using rubber-coated spheres for low-frequency bandgaps [5]. However, most of the early-developed acoustic metamaterials are still constrained by the overall structural weight, heavy resonators and narrow working bandwidth. Significant noise suppression within broadband, low-frequency range is still hard to be achieved by using lightweight structural designs.

Excitingly, as an emerging design fashion, a polymer film or thin plate-like acoustic metamaterial, with a strong structural sound-solid synthesis effect, has demonstrated great potential for prominent noise insulation, yet fulfilling lightweight demands [4,21]. Yang et al. [21] proposed the pioneering membrane-type metamaterial (MAM) in 2008, which possesses negative effective mass in the low frequency region. After that, researchers have further developed a series of lightweight membrane-type acoustic metamaterials (MAMs) and explored their excellent performance on low-frequency noise attenuation [4,22,23,24,25,26,27]. The underlying mechanism of high sound transmission loss (STL) in membrane metamaterials depends on the anti-resonance frequencies, which is determined by the resonator weight and the pretension force of the membrane. It was further observed that the sound attenuation bands in the MAMs can be enlarged with multiple resonance frequencies [24,28].

By virtue of the anti-resonance characteristics induced by the local resonance response of additional “masses,” the MAMs can break the limitations of the mass law, leading to a much higher sound insulation than mass-equally homogeneous materials. Meanwhile, the membrane-type configurations still maintain the thin and light superiorities, which are highly valued by vibration control research area. However, the existing MAMs still have some shortcomings. One of the main limitations is that the anti-resonance modes of traditional MAMs are normally generated intermittently, and such discontinuities usually lead to a narrow operating frequency band, especially in low-frequency range. How to achieve broadband low-frequency noise isolations with lightweight design is still a challenge. In addition, the attached resonators’ weight on the reported MAMs is normally heavy, while achieving lighter vibrator with broadband low-frequency sound reduction is still a pivotal aspect to improve the performance of the membrane-type meta-structures. Furthermore, most of the relevant reported research has focused on the design of membrane and resonators, rarely on the effect of resonators’ arrangement. Such an important configuration element deserves a further systematical investigation, targeting for a lighter design, broader bandwidth and more efficient attenuation.

Biomimicry, on the other hand, has been always an important innovation art and design headspring for advanced materials and structures, ranging from technology advances, built environment to medical treatment [29,30]. Inspired by nature, several bioinspired acoustic/elastic metamaterials have been proposed for pursuing good performance in vibration suppression and elastic-wave manipulation etc., such as DNA spirals metamaterials, cobweb and snowflake framework metamaterials etc. [19,31,32,33,34]. Specifically for thin film structures, the spider web design is obviously a nice candidate for the bio-fascination. A spider web has filaments distributed along the circumferential and radial directions, which can be regarded as a membrane in general. Moreover, spider webs have a series of intersecting nodes in both circumferential and radial directions, whose arrangement has a great influence on its vibration sensitivity, especially in the cases of local perturbations, such as trapped bugs. The local vibration sensitivity of the spider web topology, from some points of view, coincides with the locally-resonant design concept. The combination of the delicate natural arrangement of web-nodes and thin-films may provide better insights for the MAMs with functionalities of noise reduction, which has not been investigated, but deserves a further exploration.

In this research, we introduce the bionic configuration philosophy into the MAM design. Aiming to reduce structural weight and broaden low-frequency attenuation bandwidth, two MAM models, inspired by the spider web topology, are proposed and fabricated by polymeric membrane and attached resonators. The coupling vibration mechanisms between the bio-inspired arrangement and the host polymeric film are systematically investigated and compared with the traditional MAM configuration. The remarkable enlargements in the bandwidth of low-frequency sound attenuation, but with lighter weight design, are performed by both numerical and experimental verifications.

## 2. Structural Design and Methods

### 2.1. Spider Web-Inspired MAMs

**Figure 1:**
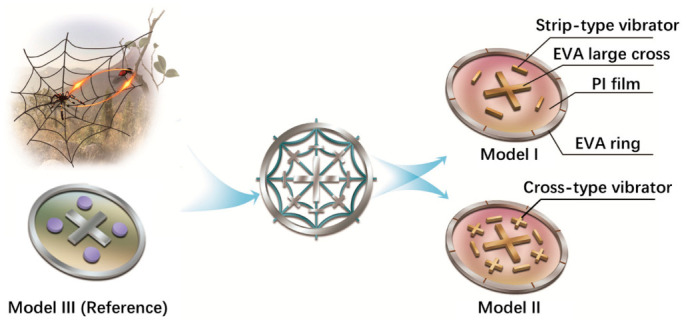
Schematic diagram of the MAM designs inspired by the spider web topology.

As shown in Figure 1, a typical spider web presents a structure with both radial and multi-layer circumferences. The spider silks distribute with an interval of intersecting nodes along both the circumferential and radial directions. Inspired by such classical distribution of the spider web knots, two kinds of MAM models, Models I and II, are investigated and compared in this research. Models I and II are designed based on the optimized bionic structure design. In addition, a reference model taken from the existing work, Model III, is selected for comparison [35]. 

In all models, a polyimide (PI) film with a thickness of 0.2 mm is fixed by a host ring structure around the circumferential boundary. The host ring is made by the ethylene vinyl acetate copolymer (EVA). A central cross, also made by EVA is positioned on the membrane center to mimic the intersecting center of the spider web. Both the EVA ring and cross structure are fabricated by 3D printing. For Models I and II, two kinds of “knots” resonators, strip-type and cross-type, are attached along the radial directions to roughly describe the radial spider web shape. The knot-like vibrators attached to the PI membrane are arranged by arch to fit the radial/circumferential direction or both at the same time, leading to a better match with the spider web characteristics.

Specifically for Model I, only strip-type resonators are attached along four radial corners to fit the circumferential direction of a spider web structure. For Model II, both strip-type and cross-type resonators are alternatively arranged along eight radial corners to fit the radial and circumferential directions simultaneously. The circle formed by the plane geometric center positions of the four “arms” is taken as the first circle of the spider web, while the position of the “knots” corresponding to the frame is applied to mimic the second circle of the spider web. For the existing reference Model III, the resonators are disc-types located at the four same radial corners of Mode I. According to the existing reference Model III, we determine the basic dimensions of the host membrane and ring structure. A further improvement is conducted by changing the resonator shape and mass with reference to the spider web, while keeping the position unchanged, but ensuring the mass is lighter than the reference model. Then, we conduct a frequency sweep and make slight dimension adjustments to achieve the target working frequencies.

**Figure A1:**
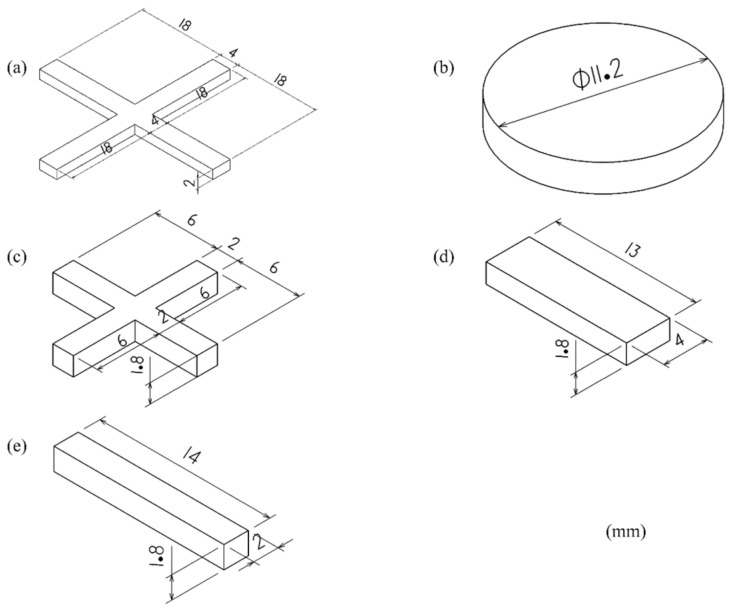
3D graphics and dimensions of the EVA cross (**a**), disc-type metal vibrator (b), small cross-type metal vibrator (**c**), large strip-type metal vibrator (**d**) and small strip-type metal vibrator (**e**). The length unit is millimeter.

For the reference Model III, the EVA ring is 2 mm thick, with an outer diameter of 100 mm and a width of 5 mm; the EVA large cross structure is 2 mm thick and the four carbon steel discs have a diameter of 1.8 mm. For Models I and II, the thickness and material of all bio-inspired vibrators are the same as the reference model III. The gravity centers of the strip-type and cross-type metal resonators in Models I and II are also the same as the disc-type metal resonators in Model III. Moreover, four small strip-type metal resonators in Model II are perpendicular to the four arms of EVA large cross structure, respectively. For the models, the radius of the circle defined by the centers of the four disc-type, strip-type or cross-type metal resonators is around 28 mm. The strip-type metal resonator and cross-type metal resonator have the same plane area of 52 mm^2^, while the plane area of the small strip-type metal resonator in Model II is 28 mm^2^. The material parameters in the numerical models are listed in Table 1, and the 3D graphics and dimensions of the structure on the membrane for the three models are shown in Figure A1 (See Appendix A). It should be mentioned that the total resonators’ weight in Model I is much lighter than that in the reference model (Model III), which is only around a half; for Model II, it is also lighter than that in Model III, around 80%.

### 2.2. Numerical Acoustic-Structure Models

**Figure 2:**
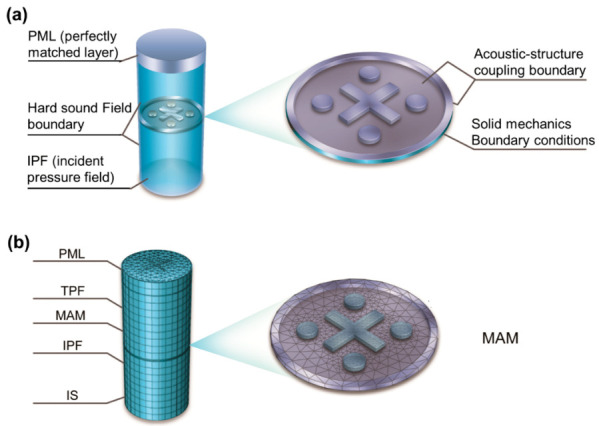
Numerical acoustic-structure models of the MAMs for (**a**) STL calculation, (**b**) finite element mesh.

A commercial FEA software, COMSOL Multiphysics (Stockholm, Sweden), is applied to establish the numerical models of the proposed MAMs, and the Acoustic-Structure Coupling module is utilized to analyze the STL characteristics. This module can describe the coupling of solid objects and three-dimensional acoustic fluid phenomena. As shown in Figure 2a, the solid mechanics part is first affected by the sound pressure to calculate the frequency response of the diaphragm, which then transmits it to the aeroacoustics domain at the other end, where an analysis is made. IS (incident surface) is the sound incident surface, IPF (incident pressure field) denotes the incident sound field, while TPF (transmitted pressure field) is the transmission sound field. PML (perfectly matched layer) is used to completely absorb the transmitted sound at the boundary to avoid boundary reflection. The air domain boundary (TPF, IPF) is set as the hard sound field boundary. A unit IPF is applied on the IS. The boundary around the MAM is set as a fixed constraint. The contact surface between MAM and TPF/IPF is set as the acoustic-structure boundary. The STL properties are calculated by the transmittance under a sweep frequency range, and the numerical model after meshing is shown in Figure 2b.

### 2.3. Experimental Setup

**Figure 3:**
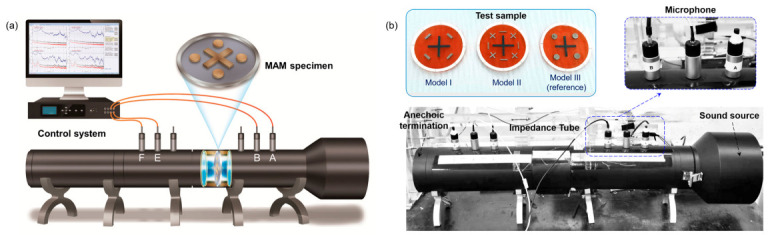
(**a**) Schematic diagram and (**b**) snapshots of the experimental setup for STL testing in acoustic impedance tube. Inset in (**b**): the fabricated samples for the three models.

Under the testing standard of ASTM E2611-09 [36], the sound isolation performance of the designed MAM models is experimentally investigated by using the acoustic impedance tube. The transmission matrix method is used to experimentally measure the transmission coefficients and the STL. The schematic diagram of experimental setup is illustrated in Figure 3a. The snapshots of prototype and test samples are shown in Figure 3b.

The four-microphone testing method with fixed positions was applied to measure the bio-inspired MAMs. The diameter of the acoustic impedance tube is 100 mm, and the measurement frequency range is from 80 Hz to 1600 Hz with a sampling interval of 0.78125 Hz. A compensation constant is applied for the material adjustment. The installation of the test piece was sealed with a rubber ring to reduce the influence of sound leakage on the transmission loss. Due to the geometric asymmetry of the experimental specimens, the incidence and reflection coefficients are not the same on both sides. Therefore, the four microphones (*A*/*B*/*E*/*F*) (see Figure 3a) of the impedance tube farthest from the specimen are selected. During testing, the direct distance between the microphone and the specimen should be far enough to ensure that the measured transfer function falls in a plane wave region. The air temperature in the tube is 24 °C; thus, the sound speed in the tube (*cs* = 346 m/s) and the wavelength (*λ*) corresponding to each measurement frequency (*f*) can be calculated.

The background noise in the testing environment was firstly measured and then subtracted from the measured result. The loudspeaker was adjusted to ensure that the measured signal amplitudes at all measuring frequencies were at least 10 dB higher than the background noise. Moreover, signal amplitudes with 60 dB lower than the maximum frequency response were also filtered out to ensure that the captured signals are as smooth as possible. Each measurement is repeated 10 times and then averaged to reduce the random errors and improve the signal-to-noise ratio. The stability evaluation under different repeated times is provided in Appendix B. The transfer function was also determined by the four-microphone method, which was corrected in advance before the formal measurement. The transfer functions were determined by the measurements from microphones A to B, E and F without specimen in the tube, respectively. Then, the positions of the two corresponding microphones were exchanged and the transfer functions were measured again. All the measurement results were used to correct the mismatch.

The STL was calculated based on the twice-measurement approach. During the first measurement, a thick sound-absorbing cotton was inserted into the end of the tube, which was removed during the second measurement. The transfer matrix can reflect the inherent physical properties of the structure and will not change with the end conditions of the pipeline. The corresponding measurement results were represented by subscripts *a* and *b*, respectively. The sound pressure *p* and particle velocity *u* on both sides of the specimen has the relationship shown in Equations (1) and (2):

(1) $${\left[\begin{array}{c} p_{1}\\ u_{1} \end{array}\right]}_{x=0}=\left[\begin{array}{cc} T_{11} & T_{12}\\ T_{21} & T_{22} \end{array}\right]{\left[\begin{array}{c} p_{1}\\ u_{1} \end{array}\right]}_{x=d}$$

(2) $${\left[\begin{array}{c} p_{2}\\ u_{2} \end{array}\right]}_{x=0}=\left[\begin{array}{cc} T_{11} & T_{12}\\ T_{21} & T_{22} \end{array}\right]{\left[\begin{array}{c} p_{2}\\ u_{2} \end{array}\right]}_{x=d}$$

where $\left[T\right]$ is the transfer matrix and x=0 and x=d denote the location coordinates of the sample’s two ends. The four intermediate parameters (IP) of the four microphones (*A*/*B*/*E*/*F*) can be calculated by using Equations (3)–(6):

(3) $$A=\frac{H_{ref,1}e^{-jkl_{1}}-H_{ref,2}e^{-jk\left(l_{1}+s_{1}\right)}}{2j\sin ks_{1}}$$

(4) $$B=\frac{H_{ref,2}e^{+jk\left(l_{1}+s_{1}\right)}-H_{ref,1}e^{+jkl_{1}}}{2j\sin ks_{1}}$$

(5) $$E=\frac{H_{ref,3}e^{+jk\left(l_{2}+s_{2}\right)}-H_{ref,4}e^{+jkl_{2}}}{2j\sin ks_{2}}$$

(6) $$F=\frac{H_{ref,4}e^{-jkl_{2}}-H_{ref,3}e^{-jk\left(l_{2}+s_{2}\right)}}{2j\sin ks_{2}}$$

where $s_{1}$ is the distance between microphone *A* and *B*, $s_{2}$ is the distance between microphone *E* and *F*, $l_{1}$ and $l_{2}$ are the distances between the reference surface (the front and back surfaces of the test piece) and microphone 2 or microphone 3, respectively, 2 and 3 refers to *B* and *E* and $H_{ref,ref}=1$ is the transfer function from the reference microphone to the *i*-th microphone.

In the STL test, the reference microphone is microphone *A*. Thus, for the reference microphone, $H_{ref,ref}=1$. The sound pressure *p* and particle velocity *u* on both sides of the specimen can be expressed by Equations (7) and (8):

(7) $$\left\{\begin{array}{c} p_{0}=A+B\\ u_{0}=\left(A-B\right)/\rho c \end{array}\right.$$

(8) $$\left\{\begin{array}{c} p_{d}=Ce^{-jkl}+De^{+jkl}\\ u_{d}=\left(Ce^{-jkl}-De^{+jkl}\right)/\rho c \end{array}\right.$$

where ρ and c are the density and sound speed, respectively. According to the above equations, the transfer matrix can be obtained by using the twice-measurement method as:

(9) $$T=\left[\begin{array}{cc} \frac{p_{0a}u_{db}-p_{0b}u_{da}}{p_{da}u_{db}-p_{db}u_{da}} & \frac{p_{0b}p_{da}-p_{0a}p_{db}}{p_{da}u_{db}-p_{db}u_{da}}\\ \frac{u_{0a}u_{db}-u_{0b}u_{da}}{p_{da}u_{db}-p_{db}u_{da}} & \frac{p_{da}u_{0b}-p_{db}u_{0a}}{p_{da}u_{db}-p_{db}u_{da}} \end{array}\right]$$

Based on the transfer matrix, the transmission coefficient can be calculated as:

(10) $$\tau_{p}=\frac{2e^{jkl}}{T_{11}+T_{12}/\rho c+\rho cT_{21}+T_{22}}$$

The relevant STL can be further written as:

(11) $$\mathrm{STL}=20lg\left|\frac{1}{\tau_{p}}\right|$$

## 3. Results and Discussion

### 3.1. STL and Anti-Resonance Modes

**Figure 4:**
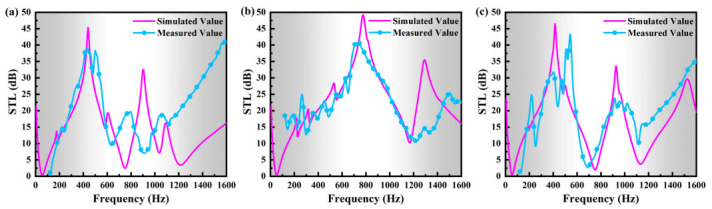
Comparison between numerically simulated and experimentally measured STL properties: (**a**) Model I, (**b**) Model II, (**c**) Model III.

Numerically calculated and experimentally measured STL properties of the three MAM models are depicted and compared in Figure 4a–c, respectively. In general, very good agreements are obtained between numerical and experimental results for all the reference and bio-inspired models, although certain derivations in both STL peaks and frequency ranges are exhibited. Overall, the experimentally measured STL peaks are slightly less than the numerical results, which is mainly induced by the manufacturing accuracy and ignoring the damping effect in numerical models. In addition, a better agreement between numerical and experimental STL bandwidths is exhibited in the low-frequency range than the high frequency range (above 1200 Hz). It can be understood because the fixed boundary condition of the ring, the bonding status between the resonators and the membrane during experimental testing, which normally have more of an effect on the high-frequency performance, cannot be guaranteed to be ideal.

**Figure 5:**
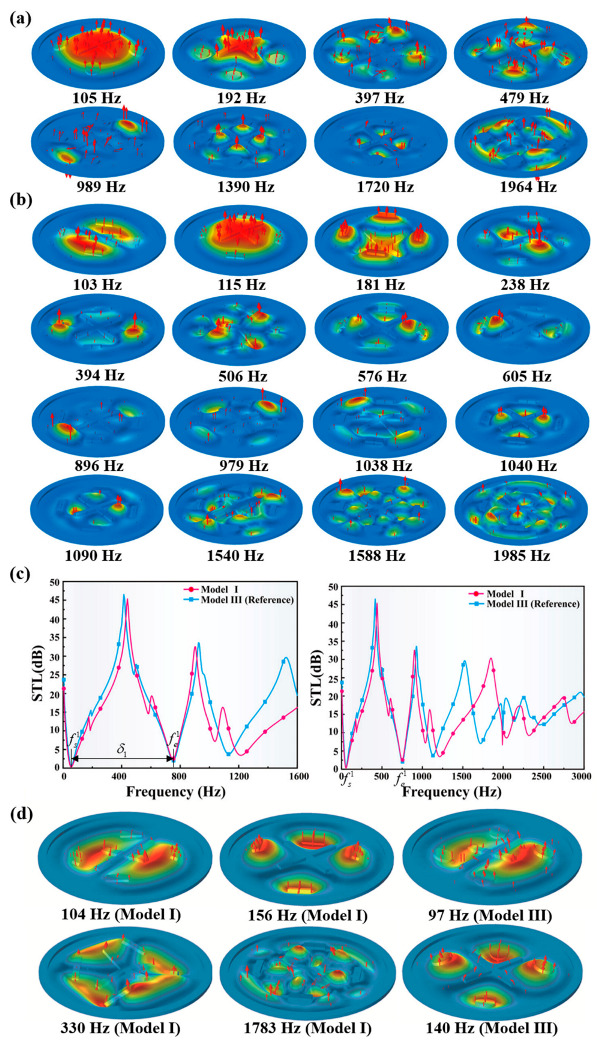
(**a**) Mode shapes of Model III (reference) at representative frequencies. (**b**) Mode shapes of Model I at representative frequencies. (**c**) Comparison between the STL profiles of the bio-inspired Model I and Model III (reference). (**d**) Mode shapes of Model I and the reference Model III at several typical frequencies.

To illustrate the sound isolation mechanisms, several mode shapes of the reference MAM model at representative frequencies are illustrated, as shown in Figure 5a. It is illustrated that within the selected low-frequency range, the reference model can generate a series of continuous, multi-state anti-resonance modes by virtue of the symmetrically distributed multi-resonators, which can keep trapping the vibration energy and maintain the dynamic balances within a relatively broad bandwidth, leading to a broadband, low-frequency sound-isolation performance. Such multi-set anti-resonance behaviors of the reference model agree with the observation in the reported research [35]. In that work, it was also demonstrated that the STL bandwidth and attenuation peaks of the reference model are greatly improvement compared to the other traditional MAM structures. However, much effort still needs to be made to achieve a better STL performance within a wider low-frequency bandwidth while using a lighter MAM design. This pivotal development aspect is the most concern in our research. 

### 3.2. Bandwidth Widening in Lighter Bio-Inspired Designs

The comparisons of sound-isolation performance between the bio-inspired and reference MAM models are systematically investigated and discussed in this section. Several typical indexes are defined to quantitatively evaluate the sound-isolation performance. The frequency range between the first and second troughs in the STL spectrum is defined as the 1st STL bandwidth, $\delta_{1}$, and the corresponding boundary trough frequencies are defined as $f_{s}^{1}$ and $f_{e}^{1}$. The normalized bandwidth for $\delta_{1}$ is defined as $\delta_{1}=\frac{\left|f_{e}^{1}-f_{s}^{1}\right|}{f_{s}^{1}}\;$.Similarly, the frequency range between the second and third troughs was defined as the 2nd STL bandwidth, $\delta_{2}$. Furthermore, in a certain frequency range of 0–1600 Hz, we define a total bandwidth as Δ to evaluate the overall performance, in which the corresponding STL is greater than 10 dB. 

The comparison between the STL profiles of the bio-inspired Model I and the reference model (Model III) is illustrated in Figure 5c. The corresponding typical indexes are listed in Table 2. Compared with Model III, Model I has almost the same STL curve for the first two STL bandwidths. The relevant normalized bandwidth $\delta_{1}^{N}$ and the total bandwidth Δ for the two models are also very similar (see Table 2). However, it is worth mentioning that the total resonators’ weight of the bio-inspired Model I is only 52.78% of that in the reference model. Excitingly, it is demonstrated that by using only a half of the weight, a nearly equal sound-isolation performance can be achieved by using the spider web-inspired design, which is a significant development in weight reduction. 

Several mode shapes of Model I at the typical frequencies are visualized in Figure 5b to explain the underlying physics. Compared with the reference model (see Figure 5d), the bio-inspired Model I has similar dipole and quadrupole modes corresponding to 104 Hz and 156 Hz. Moreover, there are also some new kinds of anti-resonance modes generated in Mode I, corresponding to 330 Hz and 1783 Hz (Figure 5d), due to the circumferential distribution of the strip-type resonators. Among them, the mode shape corresponding to 330 Hz is caused by one end of two adjacent large strip-shape vibrators, while the mode corresponding to 1783 Hz reflects the further division of the original mode by the small cross-type resonator. Such new generated modes, not shown in the reference model, maintain the broad low-frequency bandwidths even though the total resonators’ weight is reduced by half. Additionally, these multi-set responses make the STL curves of some frequency bands smoother (Figure 5c), which ensures that the bio-inspired Model I has a lighter vibrator while it processes almost the same STL curve as the reference model at low frequencies.

**Figure 6:**
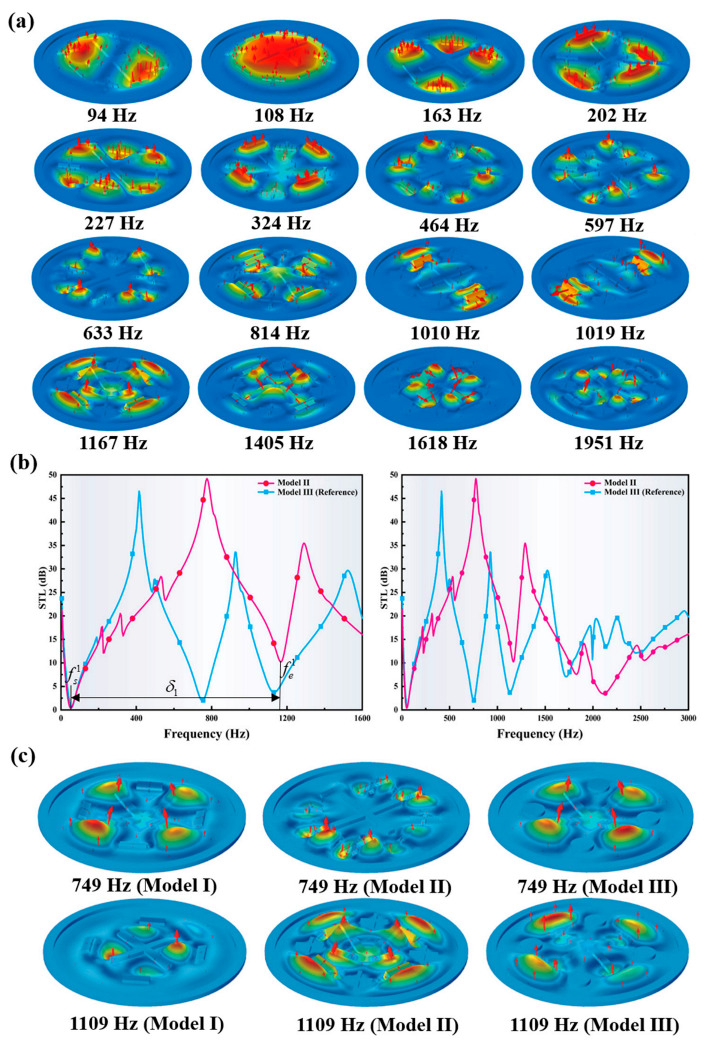
(**a**) Mode shapes of Model II at representative frequencies. (**b**) Comparison between the STL profiles of the bio-inspired Model II and the reference model. (**c**) Mode shapes of Model I, Model II and the reference Model III at several typical frequencies.

Correspondingly, the vibration mode shapes and STL properties for the bi-inspired Model II are illustrated in Figure 6a,b, respectively. Compared with the reference model (Model III), the STL bandwidth and peak value of Model II have been improved in the low frequency range (see Figure 6b). Model II has a STL peak of nearly 50 dB with a bandwidth of 1120 Hz from 50 Hz to 1170 Hz, which is 61% wider than the maximum single-peak bandwidth (695 Hz) of the reference model. Remarkably, the normalized bandwidth $\delta_{1}^{N}$ for Model II has a nearly 80% broadening than the reference model. Moreover, the total bandwidth, greater than 10 dB within 1600 Hz, is 1465 Hz for Model II, which is 26% wider than that of the reference model (1160 Hz). The remarkable total bandwidth accounts more than 91% of the sampling interval of 0–1600 Hz. Furthermore, the STL peak of Model II increases to 49.24 dB at 775 Hz, which is also higher than the reference model (46.5 dB). However, the total vibrator mass of Model II is still 19% lighter than that of the reference model.

Several mode shapes of Model II at the typical frequencies are visualized in Figure 6a to explain the underlying physics. The representative mode shapes are extracted and compared with the other modes in Figure 6c. It can be seen that the STL curves of the reference model and Model I are in the trough at the frequencies of 749 Hz and 1109 Hz, which shows similar modes and are much different from the modes of Model II at the corresponding frequencies. For the mode shapes of Model II, the four small strip-type vibrators and four cross-type vibrators merge into a shape similar to a spider web, which further divide the resonance modes of Model I, forming more local anti-resonance modes. The peaks of the STL curves generated by the new local anti-resonance modes effectively suppress the generation of troughs at similar frequencies in Model I. At the same time, more STL peaks at similar frequencies are generated by the new, continuous local anti-resonance modes. More remarkably, these adjacent peaks merge together with each other, leading to a large continuous peak in a significant wide frequency band, thus broadening the STL bandwidth and increasing the STL peak value. The bio-inspired designs allow us to demonstrate a great enhancement in sound suppression performance at a low-frequency broadband with a lighter weight.

**Figure 7:**
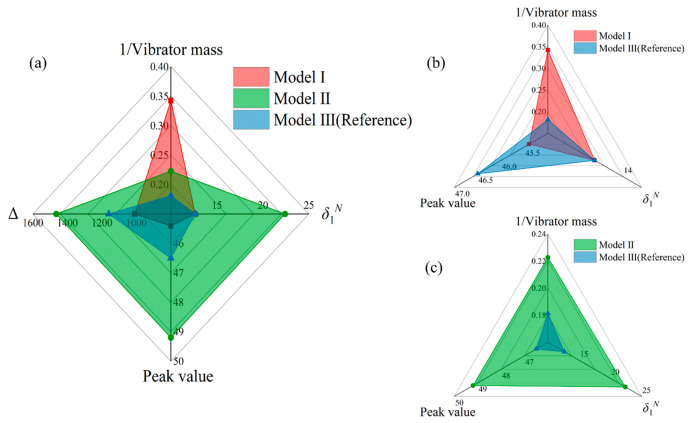
(**a**) Comparison of the overall performance between the two bio-inspired modes and the reference model. (**b**) Comparison of the overall performance between Model I and reference Model III, (**c**) Model II and reference Model III.

The comprehensive performances of the two bio-inspired models and the reference model (Model III) are further compared in the form of radar chart, as shown in Figure 7. Four typical parameters are selected to quantitatively evaluate the sound attenuation performance, as well as the structural weight.$\;\delta_{1}^{N}$ and Δ describe the bandwidth of the low-frequency sound attenuation performance. The peak value indicates the maximum STL ability, while the reciprocal of vibrator mass indicates the structural lightweight level. The reciprocal form is taken to describe the performance level uniformly. The cover area of the overall profile indicates the performance level of the sound attenuation, where the structural weight is also considered. It is evident that the higher the above parameters are, the better the comprehensive performance of the model is. It is observed that the proposed Model II covers the largest property area, which has a remarkable enhancement in the overall performance than both the reference model and Model I (Figure 7a). The sound reduction bandwidth and maximum STL performance of Model I are almost the same as that of the reference model, but its lightweight level shows a dramatic improvement (Figure 7b), which is highly desired for practical application. Model II is superior to the reference model in all dimensions (see Figure 7c). In particular, the normalized bandwidth $\delta_{1}^{N}$ in Model II is almost 180% of that in the reference model, which is a significant enhancement. These comparisons unambiguously verify the outstanding sound attenuation performance by using the bio-inspired MAM design strategies.

### 3.3. Effects of the Shape Design and Membrane Parameters

**Figure 8:**
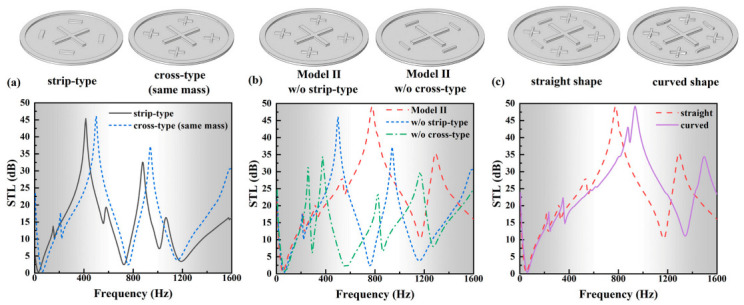
Comparison between the STL profiles of different resonator-shape designs: (**a**) strip-type and cross-type designs, (**b**) Model II without (w/o) stripe-type or cross-type, (**c**) straight and curved Model II designs.

The effects of the shape design and membrane parameters on the sound attenuation performance are further discussed. It is shown in Figure 8a that for the strip-type and cross-type resonators with the same mass and central positions, the overall trends of their STL profiles are basically same, but the peak frequencies of the cross-type design have a slight back-shift; additionally, the attenuation bandwidth has a minor enlargement. Furthermore, the influence of each design part on the bandwidth broadening in Model II is discussed. It can be observed in Figure 8b that if all stripe-type resonators are removed from Model II (only cross-type left), the main attenuation bands are around 200–700 Hz and 800–1100 Hz. If all cross-type resonators are removed from Model II, the attenuation bands are mainly around 200–500 Hz, 700–900 Hz and 1000–1200 Hz. The attenuation bands induced by the two kinds of resonators are alternately generated. However, there is no merging effect on bandwidth if only one type is left. Remarkably, when the two types are arranged together (Model II), these adjacent, alternant attenuation bands are merged together by the coupling effect, leading to a broadening band in an ultra-wide frequency range. It is demonstrated that the significant bandwidth broadening in Model II is induced by the coupling between the two types of resonators.

To make the cobweb-inspired structure more intuitive, we also constructed a curved Model II and compared its performance with the straight one, as illustrated in Figure 8c. In the curved model, all the straight bars along the circumferential direction are designed as arcs, but with the same mass as the straight one. Excitingly, the curved design presents a wider attenuation bandwidth in low-frequency range than the straight one under the same mass and positions. It is further demonstrated that the resonator shape and arrangement play a significant role in the sound attenuation bandwidth.

Furthermore, the bio-inspired Model II is employed for the sensitivity analysis of the polymeric membrane material. Three material parameters, Young’s modulus, density and Poisson’s ratio, are applied for the discussion. The parts containing EVA material in Model II include the central large cross and the frame. The frame mainly plays a fixed role and has little effect if the structural parameters are stable. Therefore, changing the material parameters mainly affects the performance of the EVA large cross. A series of EVA Young’s moduli ranging from 40% to 160% of the original value are selected for discussion.

**Figure 9:**
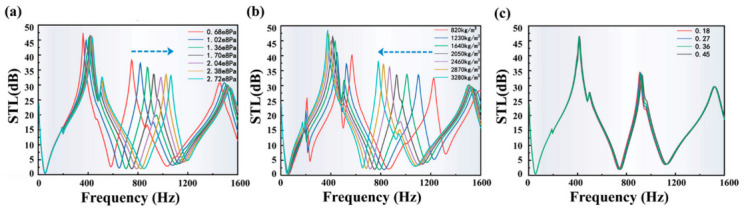
Analysis of the influence of material properties on the STL curves: (**a**) Young’s Modulus, (**b**) density and (**c**) Poisson’s ratio.

As shown in Figure 9a, when the Young’s modulus of the EVA material is changed from 0.68 × 10^8^ Pa to 2.72 × 10^8^ Pa, the 1st STL bandwidth gradually increased from 530 Hz to 795 Hz. In addition, the first low-frequency STL peak does not change too much, and the position of the second low-frequency STL peak gradually moves backward while the value gradually decreases. Therefore, in the range of Young’s modulus from 0.68 × 10^8^ Pa to 2.72 × 10^8^ Pa, the model with the maximum Young’s modulus of the EVA material presents the STL curve with the best performance.

For the analysis of the influence of density on Model II, the density of the EVA material used in this study is 2050 kg/m^3^, and 40–160% of the original EVA density was selected for calculation. As shown in Figure 9b, when the EVA density is gradually changed from 820 kg/m^3^ to 3280 kg/m^3^, the bandwidth of the 1st bandwidth gradually narrows varying from 800 Hz to 605 Hz, the first low-frequency STL peak value gradually becomes higher and the position of the second low-frequency STL peak gradually moves forward while its value gradually increases. This result is opposite to the result corresponding to the change of Young’s modulus. It is worth noting that when the density of EVA material is 820 kg/m^3^ or 1230 kg/m^3^, the first low-frequency STL peak separates. Therefore, even if the bandwidth of the first low-frequency STL peak of the low-density material is wide, it is necessary to comprehensively consider whether it is separated, because the separation would cause the first low-frequency STL peak to be far smaller than the expected one. In contrast, choosing a density of 1640 kg/m^3^ or 2050 kg/m^3^ is acceptable.

For the analysis of the influence of Poisson’s ratio on Model II, the Poisson’s ratio of the EVA material used in this study is 0.45, and 40–100% of the original Poisson’s ratio of the EVA material was selected for calculation. As shown in Figure 9c, when the Poisson’s ratio of EVA material in Model II is gradually increased from 0.18 to 0.45, the bandwidth of the first low-frequency STL peak gradually widens, but the degree of change is much smaller than the change caused by changing the same percentage of Young’s modulus and density. In addition, the change of the second low-frequency STL peak bandwidth is also very slight, indicating that the Poisson’s ratio of the membrane material has little effect on the performance of its STL curve.

## 4. Conclusions

In this paper, we propose two kinds of spider web-inspired membrane-type metamaterials for broadband low-frequency sound isolation. The bionic philosophy is combined with the design concept of acoustic metamaterials to build compact meta-structures with both prominent sound attenuation and lightweight performance. The proposed designs are fabricated by a host polymeric membrane and attached resonators. Based on the numerical and experimental investigations on the sound isolation behaviors, the following conclusions can be obtained.

1. By using the proposed bio-inspired MAM models, significant sound attenuation within a broadband low-frequency range is achieved. It is verified that the prominent attenuation performance is induced by the multi-state anti-resonance modes of the symmetrically distributed multi-resonators. Such unremitting anti-resonance behaviors can maintain the dynamic balances within a wide bandwidth by trapping the vibration energy.
2. The experimentally measured STL properties of the bio-inspired MAM structures are discussed and compared in depth. Remarkably, compared with a reference MAM model, outstanding enhancements in both attenuation bandwidth and weight-reduction performance are illustrated in the spider web-inspired designs.
3. Specifically, the bio-inspired Model I can significantly reduce the structure weight by nearly half (47%), but still maintain a same sound attenuation property as the reference model. The bio-inspired Model II can greatly enhance the comprehensive sound attenuation while keeping a lighter weight design (19% less than the reference model). The continuous attenuation bandwidth in the proposed Model II has a 61% increase compared to the reference model, while the normalized bandwidth has a significant ~80% broadening.
4. The arrangement of the spider web structure can enhance the coupling interaction between the multi-resonators and host film along the circumferential and radial directions. Therefore, more adjacent, multi-state anti-resonance modes are generated in the low-frequency range to suppress the discontinuity, leading to a broadening attenuation bandwidth.

The proposed bio-inspired MAM strategies allow us to demonstrate dramatic sound-suppression performance in both high functionality and lightweight design, which pave the way for feasible and compact sound isolation devices.

## Figures and Tables

**Table 1:** Material parameters in the membrane-type metamaterial models.

	Young’s Modulus (Pa)	Density (kg/m^3^)	Poisson’s Ratio
PI	1.42 × 10^9^	1100	0.36
EVA	1.7 × 10^8^	2050	0.45
Metal	2 × 10^11^	7800	0.33

**Table 2:** Comparison of STL properties between spider web models and the reference model.

Model	Vibrator Area (mm^2^)	Vibrator Mass (g)	STL Peak (dB)	$f_{s}^{1}\;(\mathrm{Hz})$	$f_{e}^{1}\;(\mathrm{Hz})$	$\delta_{1}\;(\mathrm{Hz})$	$\delta_{1}^{N}$	$f_{s}^{2}\;(\mathrm{Hz})$	$f_{e}^{2}\;(\mathrm{Hz})$	$\delta_{2}\;(\mathrm{Hz})$	$\delta_{2}^{N}$	Bandwidth of STL (Hz) (>10 dB)	
												Within 1600 Hz(Δ)	Within 3000 Hz
I	4 × 52	2.92	45.4	55	750	695	12.64	750	1035	285	0.38	1010	2250
II	4 × 80	4.49	49.2	50	1170	1120	22.4	1170	1825	655	0.56	1465	2360
III	4 × 98.52	5.52	46.5	55	750	695	12.64	750	1130	380	0.51	1160	2340

**Figure A2:**
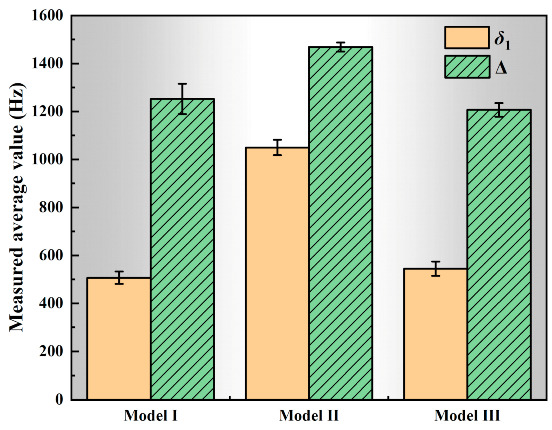
The average value of $\delta_{1}$ and Δ by measuring four samples for each model.

**Figure A3:**
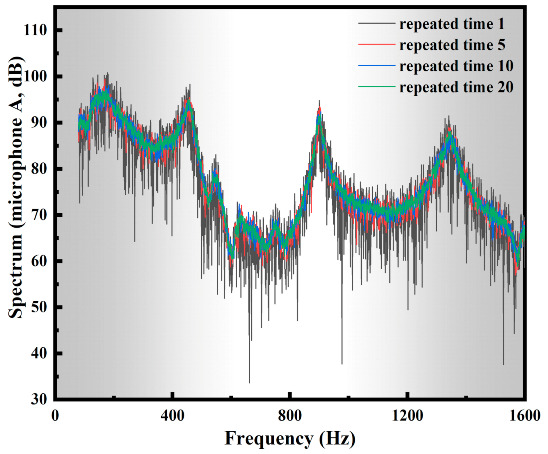
Spectrum signals captured from microphone A after various repeated times.

## Data Availability

The data presented in this study are available on request from the corresponding author.

## Appendix A

The 3D graphics and dimensions of the structure on the membrane for the three models in this paper are shown in Figure A1. The material of EVA cross and EVA frames parts is EVA, prepared by 3D printing; the material of disc-type metal vibrator, small cross-type metal vibrator, large strip-type metal vibrator and small strip-type metal vibrator is 45 steel, prepared by machining. These vibrators were assembled into all models by bonding with PI film and EVA frames.

## Appendix B

For each model, four samples are fabricated to evaluate the manufacturing and measurement accuracy. The relative errors of STL characteristics ($\delta_{1}$ and Δ) between the four tests are listed in Table A1. The measured average values with error bars for the three membrane-type metamaterial models are shown in Figure A2. It is observed that the maximum relative error range is −7.1–5.74%, which is an acceptable range for the manufacturing and measurement accuracy.

**Table A1:** The relative errors between four sample tests.

Model	STL	Test 1 (Hz)	Test 2 (Hz)	Test 3 (Hz)	Test 4 (Hz)	Relative Error (%)
I	$\delta_{1}$	505	529	523	471	−7.10~4.34
	Δ	1291	1195	1321	1202	−4.57~5.49
II	$\delta_{1}$	1049	1062	1083	1006	−4.19~3.14
	Δ	1485	1480	1442	1467	−1.80~1.12
III	$\delta_{1}$	559	507	576	537	−6.93~5.74
	Δ	1240	1187	1221	1179	−2.30~5.49

The comparison of the measured spectrums under different repeated times is conducted to check the stability. Figure A3 illustrates the spectrum signals captured from microphone A after 1, 5, 10 and 20 repeated times. It is observed that the spectrum signal has become quite stable after being repeated 10 times. Therefore, the repeated time is selected as 10 during the experimental measurement.
